# New insights into the phenotype of *FARS2* deficiency

**DOI:** 10.1016/j.ymgme.2017.10.004

**Published:** 2017-12

**Authors:** Elise Vantroys, Austin Larson, Marisa Friederich, Kaz Knight, Michael A. Swanson, Christopher A. Powell, Joél Smet, Sarah Vergult, Boel De Paepe, Sara Seneca, Herbert Roeyers, Björn Menten, Michal Minczuk, Arnaud Vanlander, Johan Van Hove, Rudy Van Coster

**Affiliations:** a Department of Pediatric Neurology and Metabolism, Ghent University Hospital, Ghent, Belgium; b Department of Pediatrics, Section of Clinical Genetics and Metabolism, University of Colorado School of Medicine, Aurora, CO, USA; c MRC Mitochondrial Biology Unit, University of Cambridge, Hills Road, Cambridge CB2 0XY, UK; d Center for Medical Genetics Ghent, Ghent University, Ghent, Belgium; e Center for Medical Genetics, UZ Brussel and Reproduction Genetics and Regenerative Medicine, Vrije Universiteit Brussel, Brussels, Belgium; f Department of Experimental Clinical and Health Psychology, Ghent University, Ghent, Belgium

**Keywords:** FARS2, Mitochondria, Mitochondrial aminoacyl-tRNA synthetase, Early-onset epileptic encephalopathy, Hereditary spastic paraplegia, Neurogenic bladder

## Abstract

Mutations in *FARS2* are known to cause dysfunction of mitochondrial translation due to deficient aminoacylation of the mitochondrial phenylalanine tRNA. Here, we report three novel mutations in *FARS2* found in two patients in a compound heterozygous state. The missense mutation c.1082C > T (p.Pro361Leu) was detected in both patients. The mutations c.461C > T (p.Ala154Val) and c.521_523delTGG (p.Val174del) were each detected in one patient. We report abnormal in vitro aminoacylation assays as a functional validation of the molecular genetic findings. Based on the phenotypic data of previously reported subjects and the two subjects reported here, we conclude that FARS2 deficiency can be associated with two phenotypes: (i) an epileptic phenotype, and (ii) a spastic paraplegia phenotype.

## Highlights

• Three novel mutations in *FARS2* were detected in two patients.
• Abnormal in vitro aminoacylation of mt-tRNA Phe was demonstrated.
• FARS2 deficiency is associated with an epileptic or a spastic paraplegia phenotype.

## 1 Introduction

Mitochondria supply most of the energy in eukaryotic cells via adenosine triphosphate (ATP) generated through the oxidative phosphorylation (OXPHOS) system located at the inner mitochondrial membrane. The OXPHOS system consists of five complexes: complex I (NADH:ubiquinone oxidoreductase), complex II (succinate:ubiquinone oxidoreductase), complex III (ubiquinol:cytochrome *c* oxidoreductase), complex IV (cytochrome *c* oxidase) and complex V (ATP synthase). Most of the subunits constituting these complexes are encoded by nuclear DNA (nuDNA) and only a minority by mitochondrial DNA (mtDNA). The OXPHOS subunits encoded by mtDNA are transcribed and translated in the mitochondrial matrix. Not only the mRNAs for those subunits but also the transfer RNAs (mt-tRNAs) and the ribosomal RNAs (mt-rRNAs) are encoded by the mtDNA. For mitochondrial transcription and translation a series of nuclear-encoded proteins are also needed [1], [2]. The latter are synthesized outside the mitochondria and subsequently imported into the mitochondrial matrix. An important group among these proteins are the mitochondrial aminoacyl-tRNA synthetases (mt-aaRSs) [3].

The mt-aaRSs are a well-described group of enzymes responsible for charging the mitochondrially-encoded tRNAs with their cognate amino acids. Defects in mt-aaRSs result in defective intramitochondrial translation, affecting the OXPHOS complexes containing mitochondrially-encoded subunits (complexes I, III, IV and V). The clinical importance of these enzymes is highlighted by the fact that all mt-aaRSs genes have now been associated with human diseases [3], [4], [5], [6], [7].

*FARS2* encodes the mitochondrial phenylalanyl tRNA synthetase. Pathogenic variants in *FARS2* have been reported so far in eighteen patients from nine different families. According to earlier publications, the reported patients had either (i) early-onset epileptic encephalopathy [8], [9], [10], [11], [12], (ii) autosomal recessive hereditary spastic paraplegia [13], [14], or (iii) juvenile onset refractory epilepsy [15].

Here, we present the clinical, biochemical and radiological findings in two unrelated patients, for whom pathogenic variants in the *FARS2* gene have been detected. Based on their clinical and radiological data new insights are gained into the phenotype of FARS2 deficiency.

## 2 Methods

### 2.1 Ethic statement

This study was approved by institutional ethical boards. Informed consent allowing further research was signed by the parents of the patients.

### 2.2 Spectrophotometric analysis

The activities of citrate synthase and OXPHOS complexes I, II, II + III, III and IV were assayed by spectrophotometric analysis in mitochondria as previously described [16], [17], [18], [19], [20], [21], [22], [23].

### 2.3 Blue native polyacrylamide gel electrophoresis

Mitochondria were isolated from skeletal muscle specimens and from cultured skin fibroblasts. The OXPHOS complexes were separated by Blue Native-Polyacrylamide Gel Electrophoresis (BN-PAGE) followed by in-gel catalytic activity staining according to previously reported methods [24].

### 2.4 Respirometry

Oxygen consumption rate in cultured skin fibroblasts derived from proband 1 was determined using a Seahorse XFp (Agilent). 35,000 cells were seeded per cell plate well and cultured for 24 h in Optimem medium (Thermo Fisher Scientific). Oxygen consumption rate was measured in triplicates in standard Seahorse assay medium (Agilent) supplemented with 10 mM glucose, 1 mM pyruvate and 2 mM glutamine (Sigma). Cells were challenged with 4 μM oligomycin, 2 μM carbonyl cyanide 4-(trifluoromethoxy)-phenylhydrazone (FCCP) and 0.5 μM of a rotenone antimycin A mix (Agilent). Afterwards, the eight well plates were collected for evaluation of protein content and citrate synthase activity per well. In each well, cells were detached after trypsinization. Protein content was assayed using Bradford reagent followed by spectrophotometric analysis. Citrate synthase activity was assayed using a method previously described by Srere, adjusted to a smaller sample volume of 200 μL [22]. Protein concentrations and citrate synthase activities were used for normalization. Spare respiratory capacity was calculated as the difference between the maximal and the basal oxygen consumption rate.

The oxygen consumption rate in cultured skin fibroblasts from proband 2 was determined on the Oxygraph2k using a modified SUIT protocol as described with the sequential addition of malate, pyruvate, digitonin, ADP, glutamate, succinate, CCCP, rotenone, antimycin A, tetramethyl-*p*-phenylenediamine (TMPD) with ascorbate, and azide [25]. Calculated parameters include acceptor control ratio (ADP-pyruvate), glutamate increase ((glutamate-ADP)/glutamate), Q-junction (succinate/glutamate), coupling ratio (succinate/CCCP), complex I (CCCP-rotenone) and complex IV (TMPD-azide). Results were compared to those of 53 runs from 29 control fibroblasts. Log transformation of ADP + pyruvate, glutamate, succinate, CCCP rates are normally distributed and results are expressed in *Z*-scores.

### 2.5 Whole exome sequencing

For proband 1, whole exome sequencing (WES) was performed using the ReliaPrep Large-Volume HT gDNA Isolation System DNA extraction kit (Promega), the SureSelectXT Human All Exome v5 (Agilent Technologies) library capture kit and a HiSeq4000 sequencer (Illumina) per standard manufacturer-recommended methods. For proband 2, WES trio sequencing with both biological parents was performed. The DNA extraction, library preparation, DNA sequencing, variant annotation and filtering were performed using previously published techniques by a commercial laboratory [26].

### 2.6 Mutation mapping

The locations of the three mutations were mapped on the crystal structure of human mitochondrial phenylalanyl-tRNA synthetase (FARS2) complexed with tRNA^Phe^ (PDB ID: 3TUP) using the program RasTop 2.2 (available at http://www.geneinfinity.org/rastop/, accessed on 7–11-17). Protein structure is shown as grey ribbon, mutation sites as red spheres, and functionally important residues as blue sticks [27]. Complexed tRNA^Phe^ is shown as orange ball-and-stick.

### 2.7 Western blot analysis

Western blot analysis was performed in mitochondrial fractions isolated from skeletal muscle. A commercial antibody against FARS2 was obtained from Proteintech Group (AB_2102499). Detection was performed by chemiluminescence (Pierce ECL plus, Thermo Fisher Scientific) per the manufacturer's instructions. Visualization was achieved using the Celvin SnapAndGo software (Biostep GmbH). The assembly of complex I was analyzed by western blotting following blue native PAGE (5–13%) of an isolated mitochondrial membrane fraction and probing with an antibody against NDUFS2, a component of the earliest complex I assembly intermediate, and compared to the results of 35 control fibroblasts, in which only a minor band at 230 kDa (< 13% of holocomplex) was observed [25].

### 2.8 Visualization of aminoacylation of four mitochondrial tRNA's

Analysis of aminoacylation status of mt-tRNAs was performed as described before [28]. RNA from patient cell lines was extracted using Trizol reagent following the manufacturer's instructions (Thermo Fisher Scientific). The final pellet was resuspended in 10 mM NaOAc (pH 5.0) and kept at 4 °C to preserve the aminoacylation state. For the deacylated control, the pellet was resuspended in 200 mM Tris-HCl at pH 9.5 and incubated at 75 °C for 5 min, followed by RNA precipitation and resuspension in 10 mM NaOAc (pH 5.0). RNA samples (5 μg) were mixed with an equal volume of sample buffer (0.1 M NaOAc pH 5.0, 8 M urea, 0.05% bromophenol blue, 0.05% xylene cyanol) and separated by PAGE (6.5%, vol/vol; 8 M urea in 0.1 M NaOAc pH 5.0 at 4 °C). RNA was blotted onto a nylon transfer membrane (Hybond, GE Healthcare). Membranes were hybridized with radioactive probes corresponding to the mt-tRNAs overnight at 65 °C in 7% SDS and 0.25 M sodium phosphate buffer (pH 7.6), washed with 1 × SSC (150 mM sodium chloride and 15 mM sodium citrate, pH 7.0) three times for 20 min and then three times with 1 × SSC containing 0.1% SDS (20 min; 65 °C), exposed to a storage phosphor screen (GE Healthcare), visualized in a Typhoon phosphorimaging system and quantified using ImageQuant software (Molecular Dynamics, GE Healthcare) or ImageJ (http://imagej.nih.gov/ij).

### 2.9 Mitochondrial translation assay

Metabolic labelling of mtDNA-encoded proteins in cultured skin fibroblasts was performed essentially as described previously [29]. Briefly, prior to metabolic labelling cells were cultured to 80% confluency in 6 well-plates in DMEM, supplemented with 10% standard FBS, 2 mM Glutamax and 110 mg/L sodium pyruvate. The medium was removed and the cells were washed twice in methionine/cysteine free DMEM, then left to incubate for 20 min at 37 °C in methionine/cysteine free DMEM supplemented with 2 mM Glutamax, 110 mg/L sodium pyruvate, 96 μg/mL cysteine and 5% dialyzed serum. To inhibit cytosolic translation, emetine dihydrochloride was then added to a final concentration of 100 μg/mL for 30 min. This allowed for specific labelling of mitochondrial translation products following addition of 100 μCi [^35^S]-l-methionine and incubation at 37 °C. After 30 min, the labelling medium was removed and cells were collected via pipetting and pelleted by centrifugation at 500*g* for 5 min. Protein lysate was resolved via SDS-PAGE, dried and imaged as described above.

## 3 Case histories and results

### 3.1 Proband 1

Proband 1 is the first-born child of healthy non-consanguineous parents. He was born at term after an uneventful pregnancy. He initially presented with poor head control at age six months. At 13 months, he could briefly sit independently. He was noted to have frequent emesis. At 15 months, he started having startle reactions followed by staring and clonic movements of the arms and legs. The echocardiogram and retinal examination were normal. Lactate in serum was increased on multiple measurements (5.1–6.4 mmol/L, normal < 2.22). Organic acid profile in urine showed an increase of lactate (16 mmol/L, normal < 4.4), pyruvate (7 mmol/L, normal < 1.1), alpha-keto-glutarate (62 mmol/L, normal < 0.7), succinate (13 mmol/L, normal < 2.6), fumarate (2.6 mmol/L, normal < 0.9) and glutarate (1.9 mmol/L, normal < 0.4 mmol/L). The amino acid profile in plasma showed an increased concentration of alanine (688 μmol/L, normal 173–440). Lactate concentration in CSF was increased (3.7 mmol/L, normal 0.9–1.9).

At the age of 19 months, he developed convulsive seizures. Carbamazepine was started as anti-epileptic treatment and in the following five months seizures did not recur. Developmentally, he never used words to communicate and lost the ability to sit without support. Clinical examination revealed lower extremity hyperreflexia at that time. Brain MRI at 20 months showed slight cortical atrophy.

At 27 months, he was admitted to hospital because of weight loss and repeated vomiting. At that time, he only had minimal head control. He also suffered from severe constipation and recurrent abdominal pain. After the age of 2 ½ years, seizures stopped except for startle myoclonus.

At eight years, he could use a wheelchair independently for mobility. He had recurrent pulmonary infections due to poor airway clearance. He developed progressive kyphoscoliosis and progressive spasticity of the lower extremities.

At 13 years, he was seen by a urologist because of bilateral cryptorchidism for which orchidopexy was performed. He was also diagnosed with a neurogenic bladder and sphincter dyssynergia. Anticholinergic medication and intermittent catheterization were started.

**Fig. 1:**
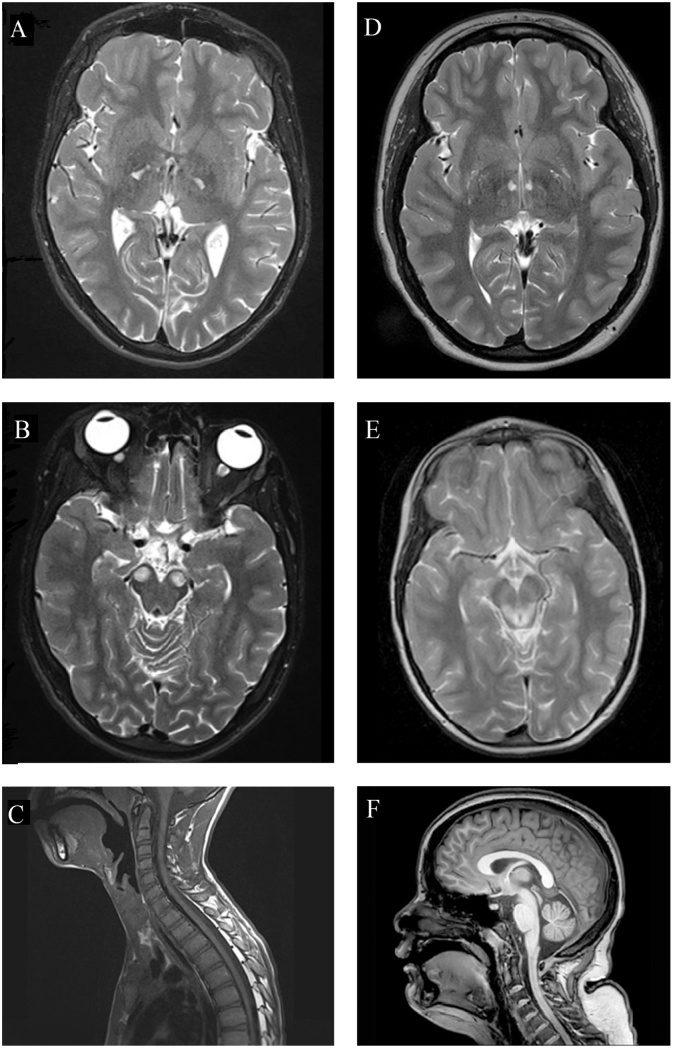
MRI studies. Brain MRI studies of proband 1 (A–C) and proband 2 (D–F). Brain MRI of proband 1 at 19 years of age showing diffuse brain atrophy (A) and T2-hyperintense lesions in the anterior medial part of the mesencephalon (B). MRI of the spinal cord of proband 1 at 14 years (C). Brain MRI of proband 2 at 15 years of age showing small lesions bilaterally in the inferior thalamic region (D). Brain MRI at 6 years with T2-hyperintense lesions of the tegmentum and the periaqueductal grey matter (E), and mild cerebellar atrophy (F).

At age 17 years, he lost the ability to use the wheelchair independently. Increasing problems with chewing and swallowing and progressively more apneic episodes were noticed. Brain MRI showed bilateral, round, focal T2-hyperintense lesions in the anterior part of the mesencephalon (frontopontine pyramidal tracts) (Fig. 1B). He underwent posterior spinal fusion for progressive scoliosis.

### 3.2 Proband 2

Patient 2 is a now 15-year-old female. She was born at 36 weeks gestation and was small for gestational age with a birth weight of 1928 g. Bilateral talipes equinovarus was noticed at birth and there was poor feeding in the neonatal period. Early gross motor milestones were delayed with achievement of independent sitting at ten months and crawling at 24 months. At 17 months, brain MRI showed symmetrical T2 hyperintensities of the posterior tegmentum. By the age of 3 ½ years, she could ambulate using a walker. At the age of six years, signs of developmental regression were noticed after an acute respiratory viral infection with loss of ambulation and development of urinary incontinence. The regression significantly affected expressive and receptive language. She also developed severe insomnia. Repeat brain MRI at that time showed more extensive T2 hyperintense lesions at the tegmentum and periaqueductal grey matter (Fig. 1E). She never regained the ability to ambulate and used an electric wheelchair for mobility after that time. She developed severe lower extremity spasticity and required femoral derotational osteotomies as well as Achilles tenotomies for management of spasticity.

At age 15 years, bradykinesia and tremor were noticed, as well as dystonic movements but no dysmetria nor ataxia. She could communicate with short phrases but with very slow and dysarthric speech. Clinically apparent seizures were never seen. MRI of the brain at age 15 years showed near resolution of the tegmental lesions but new T2 hyperintense lesions bilaterally in the anterior inferior thalamus and signs of cerebellar atrophy (Fig. 1F). Her diagnostic evaluation was significant for mild increase of lactate in serum and CSF (respectively 2.5 mmol/L and 2.9 mmol/L, normal < 2.0) and an increase of alanine in serum (762 nmol/L, normal < 547) and CSF (40 nmol/L, normal < 37). Pyruvic acid in CSF was 0.2 mmol/L (normal < 0.20) and 0.2 mmol/L in serum (normal < 0.17).

### 3.3 Biochemical and molecular studies

**Fig. 2:**
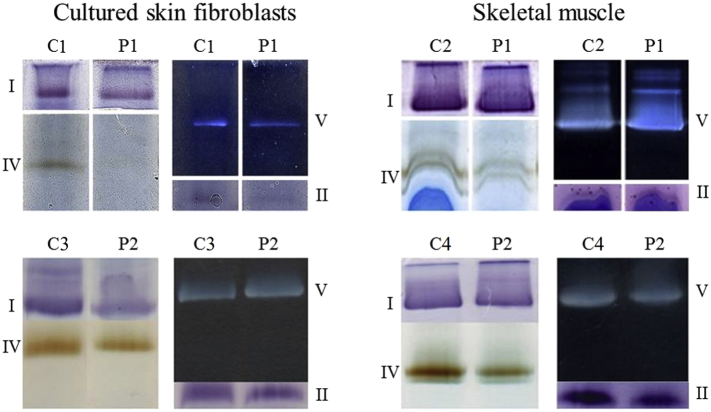
In-gel activity staining of OXPHOS complexes. In-gel activity staining of complexes I, II, IV and V in cultured skin fibroblasts and skeletal muscle of proband 1 (P1) and 2 (P2) compared to controls (C1-4). In cultured skin fibroblasts and skeletal muscle from proband 1 complex IV staining is deficient. In cultured skin fibroblasts from proband 2, complex I and complex IV staining are slightly decreased, while complexes V and II staining are comparable with the control staining. In skeletal muscle from proband 2, complex IV staining is slightly decreased, while the staining of complex I, V and II are comparable with the control staining.

**Fig. 3:**
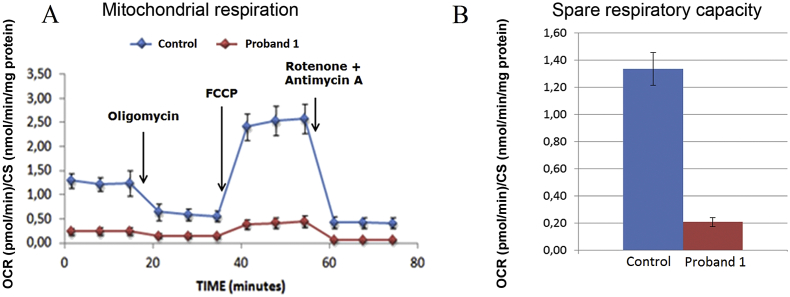
Oxygen consumption study. Oxygen consumption rate (OCR) was evaluated in cultured skin fibroblasts from proband 1 and compared to an intra-run control. OCR was normalized over citrate synthase activity (CS). Cultured skin fibroblasts of proband 1 showed a significant decrease of oxygen consumption as compared to the control during the entire run (A). The spare respiratory capacity, calculated by subtracting the basal OCR from the maximal OCR (third measurement after addition of FCCP), is significantly different between the proband 1 (0.21 ± 0.03) and intra-run control (1.34 ± 0.12) (B). The spare respiratory capacity is also significantly decreased in cultured skin fibroblasts of proband 1 when compared to a small set of inter-run controls (n = 10, 1.17 ± 0.33).

For proband 1, light microscopic examination of a skeletal muscle biopsy (*M. quadriceps* femoris) showed excessive neutral fat in muscle fibers which made interpretation of the spectrophotometric and the microscopic analysis impossible. In cultured skin fibroblasts, deficient activity of complex IV was detected (complex I not measured). During surgery, new skeletal muscle (M. *semispinalis* thoracis) and skin biopsies were taken. Spectrophotometric analysis of the second muscle biopsy showed isolated complex IV deficiency, and also in the new cultured skin fibroblasts (Table 1), confirmed by BN-PAGE followed by in-gel activity staining (Fig. 2). Determination of oxygen consumption rate (OCR) in cultured skin fibroblasts showed a severe decrease of the spare respiratory capacity when compared to controls (Fig. 3).

**Table 1:** OXPHOS spectrophotometric analyses.

Proband	Tissue	Complex I/CS	Complex II/CS	Complex II + III/CS	Complex III/CS	Complex IV/CS	Citrate synthase
Proband 1	Skeletal muscle	67 [− 1.69]	149 [− 0.14]	128 [− 0.78]	260 [− 1.71]	**337 [− 3.10]**	347
Controls (*n* = 30)		170 ± 47	197 ± 40	200 ± 46	598 ± 204	1008 ± 287	174 ± 70
Proband 1	Cultured skin fibroblasts	ND	116 [− 1.68]	168 [− 0.75]	495 [− 0.29]	**352 [− 3.80]**	94
Controls (*n* = 30)		ND	189 ± 45	233 ± 71	586 ± 165	847 ± 169	82 ± 15
Proband 2	Skeletal muscle	168 [− 0.42]	386 [0.23]	330 [0.05]	88 [0.44]	21 [1.23]	183
Controls (*n* = 22)		178 ± 52	357 ± 131	276 ± 108	70 ± 49	11 ± 6	257 ± 69
Proband 2	Cultured skin fibroblasts	**131 [− 2.04]**	682 [0.54]	412 [1.51]	59 [1.13]	8 [− 1.27]	346
Controls (*n* = 32)		229 ± 75	595 ± 175	248 ± 85	39 ± 21	12 ± 5	407 ± 112

Data are expressed as the OXPHOS activity over citrate synthase activity. Between brackets is the *Z*-score based on the logarithm of OXPHOS activity divided by the logarithm of citrate synthase activity. Control sample ratios are given as mean ± SD. *Z*-scores lower than − 1.96 are significantly different (P < 0.05) from the control samples and are considered to be deficient activities. Deficient activities are shown in bold. ND: not determined. Citrate synthase activity expressed as nanomoles of substrate per minute per milligram of protein.

mtDNA copy number and mtDNA deletions were analyzed in leukocytes and both were normal. Complete mitochondrial genome sequencing did not reveal a pathogenic variant. Therefore, WES was performed. Two missense variants were detected in *FARS2* (NM_006567.4): one in exon 2 (c.461C > T; p.Ala154Val) and one in exon 6 (c.1082C > T; p.Pro361Leu). These missense mutations were considered likely pathogenic by several in silico prediction tools (Sorting Intolerant From Tolerant [SIFT], MutationTaster, and PolyPhen-2). Heterozygous variants Ala154Val and Pro361Leu occur rarely in the population with a prevalence of 2/60,594 and 15/60,675 individuals (ExAC), respectively [30]. The two variants were confirmed to be *in trans* using targeted Sanger sequencing of the relevant DNA fragment in the parents. Western blot analysis in skeletal muscle revealed a similar amount of FARS2 protein in proband 1 when compared to two controls (data not shown).

For proband 2, a surgical muscle biopsy of the M. *quadriceps* femoris showed normal histological characteristics, normal mitochondrial ultrastructure on electron microscopy and normal staining for cytochrome *c* oxidase activity. OXPHOS complex activity in skeletal muscle was within normal limits for complexes I, II, III and IV (Table 1). BN-PAGE followed by in-gel activity staining showed normal assembly of complexes I, II, IV and V (III not assayed). Catalytic staining for complex IV was slightly decreased (Fig. 2). Spectrophotometric analysis in cultured skin fibroblasts revealed low activity of complex I. BN-PAGE followed by in-gel activity staining in cultured fibroblasts showed slightly decreased staining for complex I and IV. Complexes II and V were normal (Table 1, Fig. 2).

Trio WES was performed which showed compound heterozygosity for two variants in the *FARS2* gene (NM_006567.4): c.521_523delTGG; p.Val174del and c.1082C > T; p.Pro361Ile. The Pro361Leu variant was also found in proband 1 and Val174del is absent from ExAC [30]. The locations of the missense mutations were mapped on the published crystal structure [27]. The amino acids Ala154 and Val174 were located in the catalytic module whereas Pro361 was located in the anticodon-binding domain.

In the fibroblasts of proband 2, the oxygen consumption showed a reduced rate with pyruvate after stimulation with ADP, with glutamate, and with succinate, but low normal after uncoupling with CCCP. The calculated complex I rate was low, and the Q-point (S/G) was increased, a constellation that is typical of primarily functional complex 1 deficiency (Table 2) ([25] and Van Hove and Friederich unpublished).

**Table 2:** Oxygen consumption of fibroblasts from proband 2 in a SUIT protocol.

Parameter	Patient	Median	5th and 95th percentile
Pyruvate + ADP	12.84 (z = − 2.07) ↓	28.6	14.2–63.1
Glutamate	12.98 (z = − 2.28) ↓	30.7	15.1–65.4
Succinate	22.93 (z = − 1.85) ↓	39.3	23.0–80.3
CCCP	50.21 (z = − 1.07)	66.1	45.9–139.5
Acceptor control ratio	2.33 (z = − 0.13)	2.37	1.22–4.42
Glutamate increase	0.01 (z = − 1.85) ↓	0.08	0.01–0.13
Qpoint	1.77 ↑	1.28	1.13–1.67
Coupling ratio	0.46 (z = − 1.19)	0.61	0.34–0.83
Complex 1	26.19 ↓	48.4	31.6–123.2
Complex 4	61.50	52.3	30.8–108.2

The oxygen consumption by fibroblasts from proband 2 was evaluated in a SUIT protocol as described. Results are expressed in nmol oxygen consumed per minute and per 10^6^ cells. Following the median value, the calculated *Z*-factor is given for the variable without log transformation (glutamate increase and coupling ratio) and after log transformation (ADP + pyruvate, Glutamate, succinate, CCCP, acceptor control ratio) using mean and standard deviation derived from 53 runs of 29 control fibroblasts, which showed normality of distribution for that parameter.

### 3.4 Functional studies

**Fig. 4:**
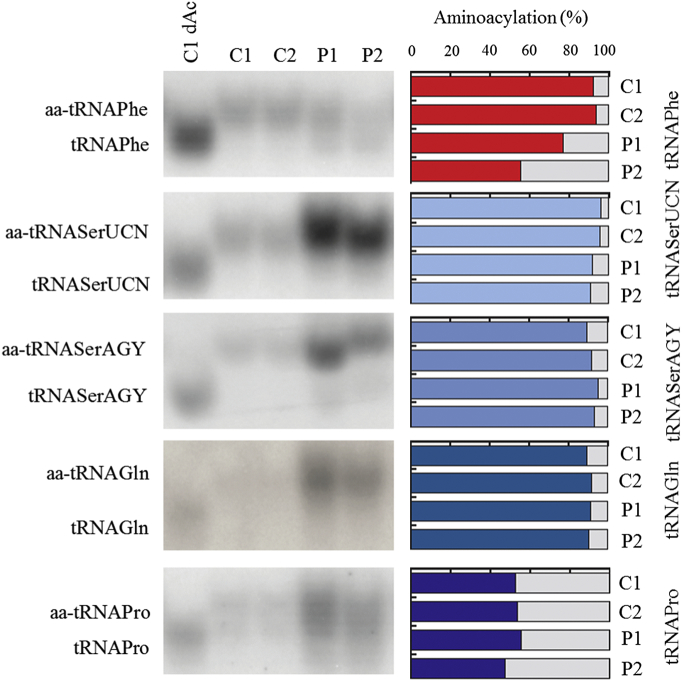
Aminoacylation status of mitochondrial tRNAs. Northern blot analysis of mitochondrial tRNA aminoacylation in total RNA samples from proband 1 (P1) and 2 (P2), and control fibroblasts (C1, C2). Membranes were hybridized with radioactive mt-tRNA probes as indicated. “dAc” indicates deacylated control sample. Densitometric analysis of the northern blot hybridized with mt-tRNA Phe shows a decrease of the ratio between aminoacylated (aa-) and deacylated form, as compared to controls. This decrease is more distinct in proband 2. A slight difference in the migration of the two mt-tRNASer products is also seen in proband 2.

**Fig. 5:**
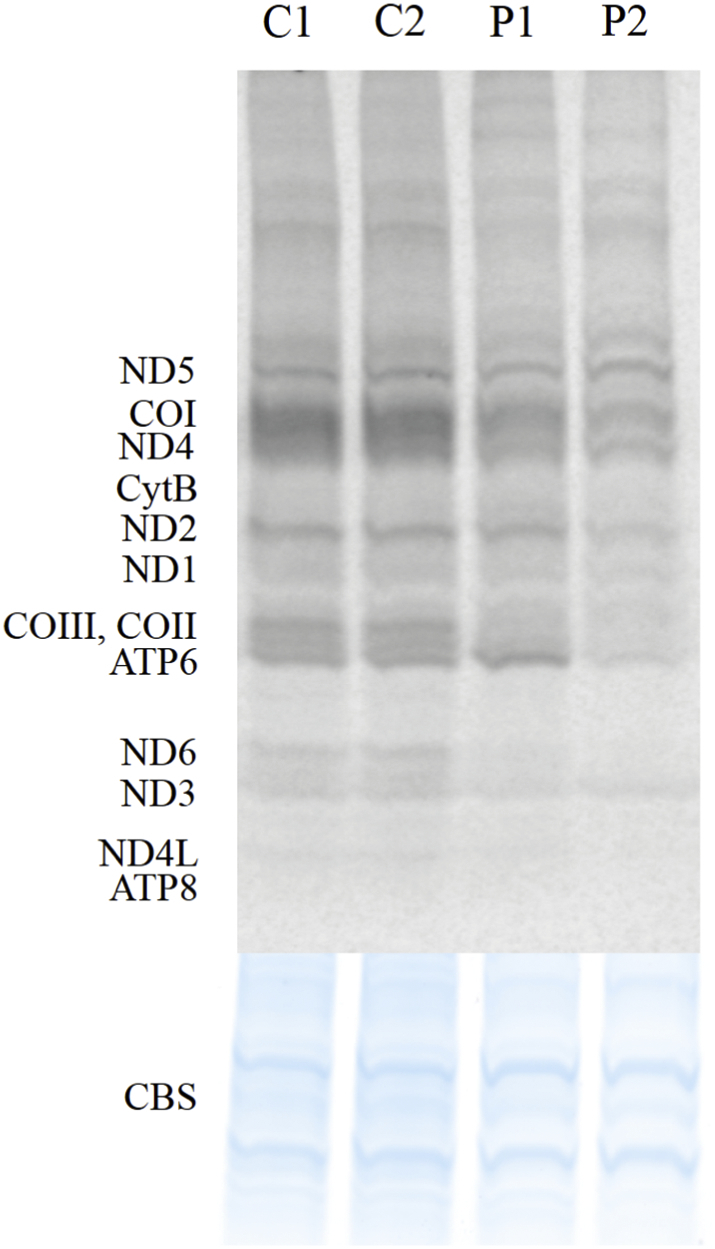
Mitochondrial translation. Mitochondrial translation in the FARS2 probands. Mitochondrial protein synthesis in cultured skin fibroblasts from proband 1 (P1) and 2 (P2), and controls (C12), was analyzed by [^35^*S*]-l-methionine pulse labelling upon inhibition of cytosolic translation by emetine. The products were resolved in SDS PAGE gel and subjected to autoradiography. mtDNA-encoded structural subunits of complex I (ND1, ND2, ND3, ND4, ND4L, ND5, ND6), complex III (CYTB), complex IV (MT-CO1, MT-CO2, MT-CO3), and complex V (ATP6, ATP8) are shown. A defect in mitochondrial protein synthesis rate is seen in both probands, with a more pronounced defect in proband 2 for some subunits (ND2, ATP6). Coomassie blue stained gel (CBS) as loading control is shown at the bottom of the picture.

Functional impact of the detected *FARS2* variants was tested by high-resolution northern blotting of RNA isolated from cultured skin fibroblasts from proband 1 and 2, allowing distinction between the aminoacyl-tRNA and the uncharged tRNA (Fig. 4). Using this approach, we consistently detected a decreased ratio between aminoacylated (aa-) and deacylated form of mitochondrial (mt-) tRNA^Phe^, while aminoacylation of control mt-tRNAs (mt-tRNA^Ser (UCN)^, mt-tRNA^Ser (AGY)^, tRNA^Gln^ and tRNA^Pro^) was normal (Fig. 4). A stronger mt-tRNA^Phe^ aminoacylation defect was observed for the proband 2. To verify whether the observed reduced availability of aa-mt-tRNA^Phe^ resulted in impairment of the mitochondrial translation rate, mtDNA-encoded polypeptides were labeled using ^35^*S*-l-methionine upon inhibition of cytoplasmic protein synthesis. A considerable decrease of translation of mitochondrially-encoded polypeptides was detected in primary fibroblasts from probands 1 and 2 (Fig. 5). The defect in mitochondrial protein synthesis rate was, for some of the subunits, stronger in proband 2, consistent with a more pronounced defect in aminoacylation of mt-tRNA^Phe^. The defect in synthesis rate was not general for all subunits of the same complex, e.g. for complex I a decreased synthesis of ND6 can be seen, while ND3 synthesis is comparable to controls.

**Fig. 6:**
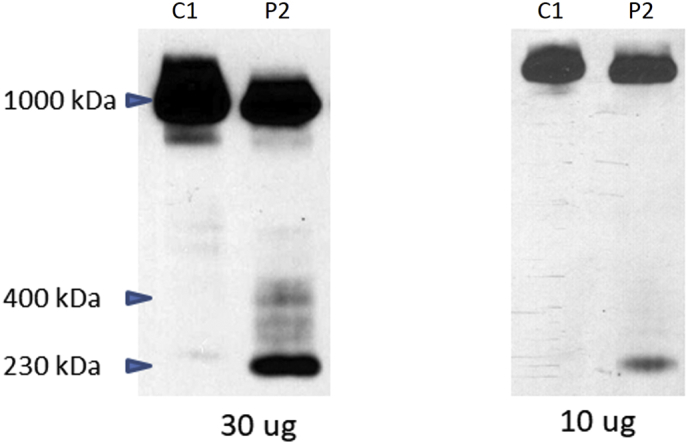
Assembly of complex I. The assembly of complex I is followed by Western blot using an antibody against NDUFS2, a component of the earliest subcomplex loading 10 and 30 μg mitochondrial membrane protein respectively. In proband 2 (P2), increased amounts are noted of the subcomplex at 230 kDa and less at 400 kDa, whereas in the control cells (C1) only a very faint band at 230 kDa is visible. This indicates a pathological slowing of the assembly of complex I in proband 2.

To investigate if the decreased translation rate of mitochondrial encoded subunits impaired the assembly of mitochondrial complexes, complex I assembly was evaluated in fibroblasts from proband 2 using an antibody against the subunit NDUFS2, which is present from the earliest subcomplexes. In normal control fibroblasts, this subcomplex was visible in 60% of cases at 7.15 ± 3.50%, maximum 13% of the fully assembled complex. In proband 2, there was an increased amount 18–28% of the subcomplex at 230 kDa (Fig. 6). The 230 kDa subcomplex is the point at which the first mitochondrial DNA encoded subunit ND1 is added to the growing complex and an increase in the 230 kDa subcomplex is observed in other defects of mitochondrial DNA translation (Friederich and Van Hove, unpublished observations).

## 4 Discussion

In previous publications, patients with FARS2 deficiency were noted to have either (i) early-onset epileptic encephalopathy [8], [9], [10], [11], [12], (ii) autosomal recessive hereditary spastic paraplegia [13], [14], or (iii) juvenile onset refractory epilepsy [15]. Here, we report two *FARS2* patients with predominantly a spastic paraplegia. When looking at the phenotypes of previously reported subjects and the two subjects reported here, we propose to define the phenotypes not based on the age of onset but rather on the predominant clinical findings, i.e. (i) an epileptic phenotype, and (ii) a spastic paraplegia phenotype, as shown in Table 3.

**Table 3:** Overview of reported *FARS2* subjects.

Subject	Citation	Mutations	Age, gender	Age at onset	Clinical phenotype	Seizures	MRI and neuropathological findings	OXPHOS activity	Functional proof of pathogenicity
1. **Epileptic phenotype**									
1	Shamseldin et al.	Y144C homozygous	22 months (deceased) F	35 days	Early-onset epilepsy, developmental delay	Myoclonic epilepsy Refractory	Severe cortical atrophy, abnormal signal intensities in putamen and nucleus caudatus (at 1.5 years)	Skeletal muscle: scattered fibers with intense NADH and SDH activity, no ragged red fibers or COX negative fibers	Yes (Elo et al.)
2	Shamseldin et al.	Y144C homozygous	< 3 months (deceased) M	/	Early-onset epilepsy, developmental delay	Myoclonic epilepsy Refractory	ND	ND	Yes (Elo et al.)
3	Shamseldin et al.	Y144C homozygous	< 3 months (deceased) M	/	Early-onset epilepsy, developmental arrest	Myoclonic epilepsy Refractory	ND	ND	Yes (Elo et al.)
4	Elo et al.	I329T D391V	8 months (deceased) F	2 days	Early-onset epilepsy, developmental arrest,	Myoclonic epilepsy, Multifocal seizures, Refractory	Severe central and cortical atrophy, slight bilateral signal abnormalities in putamina (at 3 m) Autopsy: microcephaly, almost total degeneration of frontal cortex, severe atrophy of hippocampus, basal ganglia and cerebellar cortex, Alpers syndrome	Severe complex IV deficiency in brain and skeletal muscle, Partial complex I deficiency in brain, No deficiency in cultured skin fibroblasts	Yes
5	Elo et al.	I329T D391V	21 months (deceased) F	4 days	Early-onset epilepsy, developmental delay, multiorgan failure	Refractory	ND	ND	Yes
6	Almalki et al.	D325Y microdeletion	30 months M	6 months	Early onset epilepsy, developmental delay	Infantile spasms, focal seizures, epilepsia partialis continua, prolonged seizures Refractory	Symmetrical subcortical white matter lesions. Thinning of corpus callosum	Complex IV deficiency in skeletal muscle and myoblast cell lines, Normal activities in cultured skin fibroblasts	Yes
7	Walker et al.	P85A H135D	15 years (deceased) F	/	Infantile onset motor and speech delay, refractory epilepsy, progressive neurological deterioration	Generalized tonic-clonic, focal seizures, epilepsia partialis continua, recurrent status epilepticus	Extensive signal abnormalities in left caudate, cortex and cerebellum Autopsy: almost total degeneration of occipital and frontal cortex, spongiform change in right thalamus, Alpers syndrome	No ragged-red fibers, no COX negative fibers, polarographic analysis: normal activities complex I-IV	Yes
8	Raviglione et al.	R386G microdeletion	3 years M	3 months	Early-onset epilepsy, axial hypotonia, developmental delay, strabismus, nystagmus, impaired visual fixation	Infantile spasms (3 months) Seizure free (20 months)	Microcephaly, severe brain atrophy, hyperintensity of T2 signal abnormalities in hemispheric white matter and dentate nuclei	In cultured skin fibroblasts: complex I 34% and complex IV 37% residual activity	No
9	Cho et al.	G309S homozygous	3 years M	3 months	Early-onset epilepsy, poor head control (11 months), severe motor delay, spastic legs, brisk deep tendon reflexes	Generalized tonic-clonic, myoclonic, status epilepticus	Diffuse brain atrophy	ND	No
10	Cho et al.	G309S homozygous	17 months F	4 months	Early-onset epilepsy, motor developmental delay	Myoclonic at right hand, generalized tonic-clonic, status epilepticus, Refractory	Thin corpus callosum and diffuse brain atrophy	ND	No
11	Cho et al.	G309S homozygous	8 months (deceased) M	4 months	Early-onset epilepsy	Infantile spasms, ·status epilepticus	Mild brain atrophy	ND	No
12	Cho et al.	G309S homozygous	4 months (deceased) F	3 months	Early-onset epilepsy, developmental delay	Generalized tonic–clonic, focal clonic, hemiclonic	Mild brain atrophy	ND	No
2. **Spastic paraplegia phenotype**									
13	Vernon et al.	R419C microdeletion	13 years M	First weeks	Globally delayed, truncal hypotonia, scoliosis, ptosis, Dorsal rhizotomy surgery at 5 years of age	Complications in neonatal period, seizures only in the neonatal period	Two foci of signal abnormalities in periventricular and deep white matter of right posterior frontal lobe (13 years)	ND	No
14	Vernon et al.	R419C microdeletion	5 years F	2 months	Globally delayed, strabismus, truncal hypotonia, bilateral equinovarus, brisk deep tendon reflexes, intention tremor, dysarthric speech	One seizure at 2 months of age	Normal (at 3 years)	ND	No
15	Yang et al.	D142Y homozygous	41 years F	2 years	Progressive limb spasticity, pyramidal weakness, hyperreflexia, extensor plantar reflexes, scissors gait	No seizures	Normal	ND	Yes
16	Yang et al.	D142Y homozygous	30 years M	1 year	Same phenotype as 15	No seizures	Normal	ND	Yes
17	Yang et al.	D142Y homozygous	26 years F	5 years	Same phenotype as 15	No seizures	Normal	ND	Yes
18	Yang et al.	D142Y homozygous	23 years F	3 years	Same phenotype as 15	No seizures	Normal	ND	Yes
19	Proband 1	A154V P361L	19 years M	6 months	Developmental delay, spastic paraplegia, neurogenic bladder	Mild seizures only between 15 and 30 months of age	Signal abnormalities in anterior parts of mesencephalon Diffuse brain atrophy	Complex IV deficiency in skeletal muscle and cultured skin fibroblasts	Yes
20	Proband 2	V174del P361L	15 years F	10 months	Delayed motor development, spastic paraplegia	No seizures	Signal abnormalities in tegmentum and periaqueductal grey matter Mild progressive cerebellar atrophy	Complex I deficiency and low activity of complex IV in cultured skin fibroblasts Normal activities in skeletal muscle	Yes

The epileptic phenotype of FARS2 deficiency is the most severe phenotype. Delayed motor development is seen in the first six months of life. All of them (12/12) suffered from epilepsy, in most of them starting in the first years of life. Brain MRI was abnormal in all the subjects in whom MRI was performed (9/9) and showed a wide range of abnormalities including diffuse brain atrophy, cerebral cortical atrophy, thin corpus callosum, atrophic cerebellum, lesions in dentate nuclei and mesencephalon, as well as signal abnormalities in putamen, caudate nucleus and white matter. More than half of the subjects (7/12) with the severe phenotype died before the age of two years. The spastic paraplegia phenotype is less severe. It was first reported in four siblings born from consanguineous parents [14]. The two siblings reported here can also be classified as having the spastic paraplegia phenotype. Proband 1 suffered from mild seizures between the age of 15 and 30 months but afterwards his clinical picture was dominated by spastic paraplegia. Proband 2 also had spastic paraplegia, no seizures. Also, the two subjects reported by Vernon et al. [13] can be categorized into the spastic paraplegia phenotype. One of them was a 5-year-old girl who had only one seizure at the age of two months and afterwards was found to have spastic paraplegia. Her older brother had neurological complications in the neonatal period accompanied by seizures. Later, he did not present anymore with seizures but underwent dorsal rhizotomy surgery at the age five years which is a surgical procedure typically performed for spastic paraplegia/tetraplegia.

The neuroradiological findings in proband 1 were unusual. Cerebral MRI showed prominent round signal abnormalities bilaterally in the most anterior parts of the mesencephalon. Only one other subject was reported in the literature with similar lesions; that subject had ECHS1 deficiency [31]. Brain MRI in proband 2 showed signal abnormalities in the posterior part of the mesencephalon around the aqueduct, a well-described finding in patients with mitochondrial disease [32].

In the probands presented here, there was a long delay in determination of the molecular genetic diagnosis despite the early characteristic features of mitochondrial disease. The regression in both probands and the increased lactate concentrations in serum, urine and CSF, as well as the increased concentrations of alanine in serum and the increase of Krebs cycle intermediates in urine was highly suggestive of a mitochondrial defect. It was only after the use of WES that we could pinpoint the underlying molecular defect. In the future, more subjects with a FARS2 defect will likely be detected as WES continues to be more frequently used as diagnostic tool in the clinical setting.

**Fig. 7:**
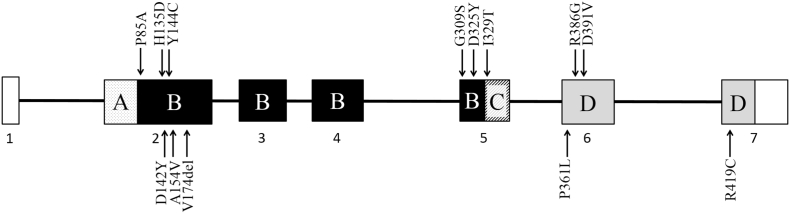
Overview of reported *FARS2* mutations at transcript and protein level. Location of reported FARS2 mutations at the transcript and protein level. Mutations associated with the epileptic phenotype are shown in the upper side of the figure, mutations associated with the spastic paraplegia phenotype in the lower part. Transcript NM_006567.4 consists of 7 exons of which 6 are coding (exon 2–7). Almost all mutations were found in exon 2, 5 and 6. The protein structure of FARS2 consists of four domains: (A) the N-terminal region (residues 36–83, dotted pattern), (B) the catalytic domain (residues 84–325, black), (C) the linker region (residues 326–358, hatch pattern) and (D) the anticodon binding domain (residues 359–451, grey). Eight of the thirteen mutations were found in the catalytic domain, four in the anticodon binding domain and one in the linker region. No mutations have been found in the N-terminal region. The two probands presented here are compound heterozygous for a mutation in the anticodon binding domain (Pro361Leu) and one in the catalytic domain (Ala154Val and Val174del).

WES identified compound heterozygotes for missense mutations in the two probands described here. Both probands are compound heterozygous for a mutation in the anticodon binding domain (Pro361Leu) and one in the catalytic domain (Ala154Val and Val174del) confirming the association of both the catalytic and anticodon binding domain with a severe phenotype. Most of the pathogenic variants reported in literature are in exon 2 of the *FARS2* gene (6/13) and some in exon 5 and 6 (3/13 in each) (Fig. 7). Only one pathogenic variant was reported in exon 7. FARS2 protein consists of 4 domains: the N-terminal region (residues 36–83), the catalytic domain (residues 84–325), the linker region (residues 326–358) and the anticodon binding domain (residues 359–451) [33]. No clear link can be detected between the domain affected by the mutation and phenotype. Most mutations are in the catalytic domain and homozygous mutations in this domain can cause both the epileptic (Tyr144Cys) as the spastic paraplegia phenotype (Asp142Tyr). The epileptic phenotype has also been described with mutations in the catalytic domain, anticodon binding domain and linker region. So far, no mutations have been reported in the N-terminal region.

**Fig. 8:**
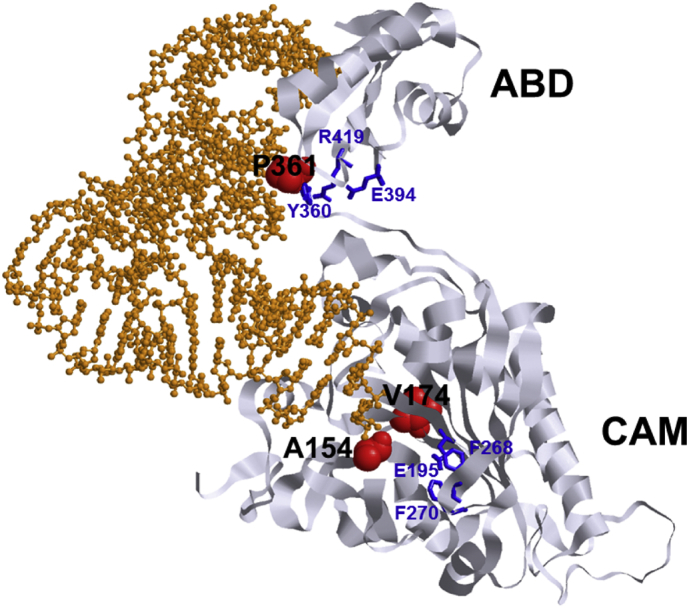
Location of mutations in the FARS2 protein. The location of mutated amino acids Ala154, Val174 and Pro361 mapped on the crystal structure of human mitochondrial phenylalanyl-tRNA synthetase complexed with tRNAPhe (PDB ID: 3TUP). Mutated amino acids are shown as red spheres. Structurally important amino acids (Tyr360, Glu394 and Arg419) involved in the stabilization of the anticodon-binding domain (ABD) and catalytically important amino acids (Glu195, Phe268 and Phe270) in the active site of the catalytic module (CAM) are shown as blue sticks [27]. Complexed tRNAPhe is shown as orange ball and stick.

Molecular modeling showed the locations of Ala154, Val174 and Pro361 mapped on the crystal structure of human mitochondrial phenylalanyl-tRNA synthetase FARS2 complexed with tRNA^Phe^ (PDB ID: 3TUP) (Fig. 8). Two of the mutations sites (Ala154 and Val174) are located near the active site in the catalytic module (CAM) of FARS2 protein. Ala154 is located in the active site pocket. The valine mutation at this site could potentially change the shape of the pocket resulting in the inability of substrate phenylalanine from interacting with Phe268 and Phe270, which are responsible for substrate recognition [27]. Val174 is located in a β-sheet adjacent to the active site Glu195 which participates in hydrogen bonding with the phenylalanine substrate and transition state intermediates through a coordinated water molecule [27]. The amino nitrogen of Val174 is 5.18 Å from the carbonyl oxygen of Glu195. An alanine substitution at this position could result a change in the orientation of the Glu195 and the inability to properly coordinate the phenylalanine substrate in the active site. Pro361 is located in the anticodon binding domain (ABD) of the FARS2 protein and is right next to Tyr360 which participates in hydrogen bonding interactions with Glu394 and Arg419. The Arg419 residue has been described as shape-generating for the ABD and the interactions with Tyr360 and Glu394 are important for the structural stabilization of the ADB [27]. The Pro361Leu mutation could lead to a structural change that breaks the hydrogen bond between Tyr360 and Arg419 which would affect the stability of the ABD leading to decreased interaction with the tRNA^Phe^.

Although the amino acids involved are conserved and in silico programs predict pathogenicity, and molecular modeling suggests that mutating these amino acids may affect enzymatic function, direct functional testing is required to confirm the pathogenicity of the findings. FARS2 catalyzes the charging of the tRNA^Phe^, and in the two probands presented here we demonstrated a decreased amount of Phe-charged tRNA and an increased amount of deacylated tRNA for phenylalanine. We next documented that this reduced charging affected mitochondrial translation in a direct assay using labeled methionine. Next we evaluated whether the decreased mitochondrial translation affected the assembly of the OXPHOS complexes, and documented abnormal assembly intermediates of complex I.

Microscopic and histochemical examination of skeletal muscle initially did not reveal abnormalities suggestive of a mitochondrial defect in proband 1 and proband 2 (no ragged-red fibers, no COX negative fibers). This was also the case in previously reported subjects with FARS2 deficiency [8], [15]. In proband 1, biochemical investigations in skeletal muscle and in cultured skin fibroblasts revealed a complex IV deficiency. In proband 2, OXPHOS complex activities were normal in skeletal muscle, while a complex I deficiency was found in cultured skin fibroblasts. This is peculiar since skeletal muscle is usually more affected than cultured skin fibroblasts. These results suggest that the skeletal muscle is less affected in proband 2 which correlates well with the clinical findings. Proband 2 ambulated until the age of six years while proband 1 never gained the ability to walk independently. In previously reported FARS2 deficient subjects, biochemical studies are scarce. Biochemical investigations in skeletal muscle were reported in only three subjects. In two of them an isolated complex IV deficiency was found and in one a normal activity of the OXPHOS complexes I to IV [9], [10], [15]. Biochemical investigations in cultured skin fibroblasts were reported in only three subjects. In two of them, the OXPHOS activities were normal and in the other a decrease of the activities of complex I and IV was detected [9], [10], [11]. The conclusion is that the OXPHOS defects detected in FARS2 deficient subjects vary from normal, to isolated complex IV deficiency, isolated complex I deficiency, or to a combined complex I + IV deficiency. The variable involvement of intra-mitochondrial translation preferentially affects complexes I and IV, as these are the two complexes that are composed of the largest number of mitochondrially-encoded subunits.

Abnormal subcomplexes of complex V are expected to be seen in mitochondrial translation defects when using Blue Native PAGE. They were not seen in either proband in this report, indicating that there is still enough translation of the two complex V subunits. On oxygen consumptions studies, proband 1 had a decrease in the spare respiratory capacity. Proband 2 had decreased complex 1 activity and increased Q-point (succinate/glutamate ratio), indicative of reduced effective complex I rate. The variable enzymatic test results in the patients with FARS2 deficiency highlight the difficulties in recognizing the mitochondrial dysfunction using traditional enzymatic methods in cultured skin fibroblasts. In known patients with mitochondrial disorders, fibroblasts only detect an enzymatic deficiency in half of the patients [34], illustrating the need for more targeted testing. As shown here, targeted functional testing based on the identified genetic defect of tRNA charging, mitochondrial translation and complex I assembly could clearly identify functional defects, even in fibroblasts. This case illustrates the complementary nature of broad molecular genetic testing and targeted functional analysis of mitochondrial enzymes in the diagnostic evaluation of the patients with suspected mitochondrial disease, even when using fibroblasts as tissue for functional analysis, which can be obtained from a minimally invasive skin biopsy.

In conclusion, we report two patients with three different mutations in *FARS2* as well as indirect functional evidence of likely pathogenicity of the mutations. Both subjects had spastic paraplegia and can be added to the group of patients with predominantly spastic paraplegia. We can conclude that FARS2 deficient subjects can be categorized into either an epileptic phenotype or a spastic paraplegia phenotype.

## Funding

This work was supported by the support fund Marguerite-Marie Delacroix and the Special Research Fund (BOF) from the Ghent University (grant number BOF.24J.2014.0005.02) (EV). CAP and MM were supported by the Medical Research Council (MRC) (MC_U105697135), UK core funding for the MRC Mitochondrial Biology Unit, University of Cambridge (MC_U105697135). SV was supported by a postdoctoral fellowship of the Fund for Scientific Research Flanders (FWO). JVH, MFW, KK, and AL were supported by Miracles for Mito, Summits for Samantha and the Children's Hospital Colorado Foundation.
